# Integrated Assessment of Metabolic, Oxidative, and Molecular Adaptations from Pregnancy to Early Lactation in Shami Goats (*Capra hircus*)

**DOI:** 10.3390/vetsci13080780

**Published:** 2026-08-04

**Authors:** Haifa Ali Alqhtani, Tahani M. I. Al-Hazani, Ahmed El Sayed, Ahmed Ateya, Ahmed H. Ghonaim, Rowa K. Zarah, Fatmah A. Safhi, Adel Almubarak, Hussein Babiker, Rasha yassin Elkhidr, Wael M. El-Deeb, Ahmed Magzoub Khalid, Mayyadah Abdullah Alkuwayti, Mohamed Marzok

**Affiliations:** 1 Department of Biology, College of Science, Princess Nourah bint Abdulrahman University, P.O. Box 84428, Riyadh 11671, Saudi Arabia; haalqhtani@pnu.edu.sa (H.A.A.); faalsafhi@pnu.edu.sa (F.A.S.); 2 Biology Department, College of Science and Humanities, Prince Sattam bin Abdulaziz University, P.O. Box 83, Al-Kharj 11940, Saudi Arabia; t.alhazani@psau.edu.sa; 3 Department of Animal Health and Poultry, Animal and Poultry Production Division, Desert Research Center (DRC), Mataryia, Cairo 11753, Egypt; a.ghonaim@webmail.hzau.edu.cn; 4 Department of Development of Animal Wealth, Faculty of Veterinary Medicine, Mansoura University, Mansoura 35516, Egypt; dr_ahmedismail@mans.edu.eg; 5 National Key Laboratory of Agricultural Microbiology & Hubei Hongshan Laboratory, Huazhong Agricultural University, Wuhan 430070, China; 6 Department of Biology, College of Science, Taif University, Taif 21944, Saudi Arabia; rowa.z@tu.edu.sa; 7 Department of Clinical Sciences, College of Veterinary Medicine, King Faisal University, P.O. Box 400, Al-Ahsa 31982, Saudi Arabia; aimubarak@kfu.edu.sa (A.A.); hbabiker@kfu.edu.sa (H.B.); relmola@kfu.edu.sa (R.y.E.); weldeeb@kfu.edu.sa (W.M.E.-D.); 8 Department of Veterinary Public Health, College of Veterinary Medicine, King Faisal University, P.O. Box 400, Al-Ahsa 31982, Saudi Arabia; amkali@kfu.edu.sa; 9 Department of Biological Sciences, College of Science, King Faisal University, Al-Ahsa 31982, Saudi Arabia; malkwaiti@kfu.edu.sa

**Keywords:** Shami goats, periparturient period, metabolic adaptation, oxidative stress, gene expression, reproductive physiology

## Abstract

Identifying physiological changes during the transition period is essential for improving the health and productivity of dairy goats. This study evaluated hematological, biochemical, hormonal, oxidative stress, and molecular alterations in Shami goats during the pre-pregnancy, late pregnancy, and early lactation periods. Eighty clinically healthy goats were examined, and blood samples were analyzed for hematological indices, metabolic and hormonal profiles, oxidative stress biomarkers, and relative expression of genes associated with energy metabolism, antioxidant defense, inflammation, and autophagy. Late pregnancy was characterized by significant (*p* < 0.05) increases in red blood cell count (RBCs), hemoglobin concentration (Hb), neutrophils, albumin, globulin, urea, insulin-like growth factor-1 (IGF-I), and malondialdehyde (MDA), accompanied by decreased glucose, cholesterol, total protein (TP), antioxidant markers, total leukocyte count, packed cell volume, and monocytes. Early lactation was associated with higher non-esterified fatty acid, triiodothyronine (T3), and thyroxine (T4) levels. Genes involved in lipid mobilization and oxidation, ketogenesis, inflammation, cellular stress, and autophagy; *sirtuin 1 (SIRT1)*, *peroxisome proliferator-activated receptor alpha (PPARA)*, *carnitine palmitoyltransferase 1a (CPT1A)*, *3-hydroxy-3-methylglutaryl-coenzyme a synthase 2 (HMGCS2)*, *cluster of differentiation 36 (CD36)*, *lipase E (LIPE)*, *protein kinase amp-activated catalytic subunit alpha 1 (PRKAA1)*, *solute carrier family 2 member 1 (SLC2A1)*, *haptoglobin (HP)*, *interleukin 6 (IL6)*, *heat shock protein 70 (HSP70)*, *heme oxygenase 1 (HMOX1)*, *beclin 1 (BECN1)*, and *autophagy-related protein 5 (ATG5)* were significantly upregulated, whereas antioxidant- and glucose transport-related genes *nuclear factor erythroid 2-related factor 2 (Nrf2)*, *glutathione peroxidase 1 (GPX1)*, *catalase (CAT)*, *thioredoxin (TXN)*, and *solute carrier family 2 member 4 (SLC2A4)* were downregulated during the transition period. The results indicate well-orchestrated metabolic and molecular adaptations that can be employed as biological indicators to track the physiological status of Shami goats. These findings fulfilled the study objective and identified potential biomarkers of the transition period in Shami goats.

## Simple Summary

Pregnancy and early lactation are the most physically taxing stages in the reproductive cycle of dairy goats, necessitating significant metabolic, endocrine, immunological, and molecular adjustments to facilitate fetal development and milk synthesis. This study evaluated 80 Shami goats during the pre-pregnancy, late pregnancy, and early lactation periods by analyzing their hematological, metabolic, hormonal, antioxidant, and gene expression profiles. Late pregnancy was characterized by increased red blood cell count, hemoglobin concentration, neutrophil, albumin, globulin, urea, insulin-like growth factor-1, and malondialdehyde levels. Conversely, the white blood cell count, packed cell volume, glucose, cholesterol, total protein, and antioxidant markers declined. Early lactation was associated with increased non-esterified fatty acid, triiodothyronine, and thyroxine concentrations. Genes involved in lipid mobilization, fatty acid oxidation, ketogenesis, inflammation, cellular stress, and autophagy (including *SIRT1*, *PPARA*, *CPT1A*, *HMGCS2*, *CD36*, *LIPE*, *PRKAA1*, *HP*, *IL6*, *HSP70*, *BECN1*, and *ATG5*) were progressively upregulated, whereas antioxidant defense and insulin-dependent glucose transport genes were downregulated. These biomarkers may facilitate the monitoring of reproductive health and enhance transition management in dairy goats.

## 1. Introduction

Goats play an important part in the production sector of livestock, especially in the arid and semi-arid areas, where they help with food security and the livelihoods of the people [1]. In Egypt, the goat population is estimated at approximately 1.140 million head in 2024 [2], reflecting their economic importance in the national livestock industry. Although goats produce less milk than cattle and buffaloes, consumer demand for goat milk and its dairy products, particularly cheese, has increased steadily because of their nutritional and health benefits [3]. Among the various goat breeds, the Shami (Damascus) goat is recognized for its superior productive and reproductive performance [4,5]. This breed exhibits remarkable adaptability to diverse climatic conditions, especially the harsh environments of arid and semi-arid regions [6]. Owing to its great genetic potential for both milk and meat production, the Shami goat is considered a valuable dual-purpose breed [7]. Consequently, it has been widely incorporated into genetic improvement and crossbreeding programs intended to augment the productivity of indigenous goat breeds in several countries [8].

The nutritional requirements of goats vary according to their physiological stage [9]. Among these phases, the periparturient period, which encompasses late pregnancy and early lactation, is one of the most serious phases due to the profound metabolic and physiological adaptations essential to maintenance, parturition, and the onset of milk production [10]. During this transition, animals undergo marked changes in feed intake, endocrine regulation, and nutrient partitioning to meet the cumulative demands of fetal growth and lactogenesis [11]. However, reduced dry matter intake coupled with elevated energy requirements frequently results in a state of negative energy balance, predisposing animals to immunosuppression and impaired health [12].

Metabolic stresses that arise during the transition period are also factors in increasing the likelihood of developing diseases like pregnancy toxemia, fatty liver disease, hypocalcemia, and hypomagnesemia [13,14]. In order to compensate for the energy shortfall, the fats in the body become mobilized, resulting in increased levels of non-esterified fatty acids (NEFA) and β-hydroxybutyric acid (β-HBA), both of which are considered markers of negative energy balance [15]. Moreover, significant alterations occur in several blood biochemical parameters, including glucose, insulin, total protein (TP), cholesterol, triglycerides, and creatinine, reflecting the metabolic adjustments during this period [16]. Therefore, monitoring these metabolic biomarkers is essential for the early detection of metabolic disturbances, enabling timely intervention and reducing economic losses in goat production systems [17].

Acute-phase proteins (APPs), which are mainly produced in the liver, are useful markers for determining the health status and diagnosing various diseases in animals [18]. There are many variables that influence the concentration of APPs, including infectious and non-infectious diseases and physiological states such as nutrition, pregnancy, and lactation, along with environmental stressors [19]. Hence, monitoring APP levels in different physiological states may help understand the health status, immune response, and reproductive efficiency in animals [20].

Transition periods also result in oxidative stress due to an imbalance between the excess formation of reactive oxygen species (ROS) and the antioxidant system [21]. Under normal physiological conditions, animals possess efficient antioxidant mechanisms that neutralize free radicals and maintain redox homeostasis [22]. Among these defense mechanisms, enzymatic antioxidants, particularly catalase, play a crucial role in decomposing hydrogen peroxide into water and oxygen, thereby protecting cells from oxidative damage [23]. Hence, the examination of both APPs and biomarkers of oxidative stress offers a thorough assessment of the physiological and metabolic adjustments that take place during critical production periods.

Understanding the physiological and molecular reactions of animals to stress during different production phases is important for making them more adaptable and productive [24,25]. Genetic selection for greater adaptation to harsh environments has become a major priority in livestock research [26]. Identifying variations in the expression of genes related to immune regulation, inflammation, and stress response is useful for understanding the molecular mechanisms underlying adaptation [27]. Such genes may serve as prospective biomarkers for selecting resistant animals in breeding programs [28].

Although the Shami goat is economically significant, there are no data on the physiological and molecular processes that occur during the critical period of transition from pregnancy to lactation. In particular, data on acute-phase proteins, metabolism, oxidative stress biomarkers, and the expression of the corresponding genes are limited. Therefore, the objective of this study was to investigate the alterations in acute-phase proteins, metabolic profiles, oxidative stress biomarkers, and the expression of selected genes associated with inflammation and stress responses in Shami goats during the pre-pregnancy, late pregnancy, and early lactation stages.

## 2. Materials and Methods

### 2.1. Animals and Reproductive Experimental Design

A total of 80 apparently healthy, non-pregnant, non-lactating Shami does aged 5–7 years (6.1 ± 0.6 years) and weighing 33–49 kg (40.9 ± 5.1 kg) were enrolled in this study. This study was conducted on a private farm in Al-Ahsa Governorate, Saudi Arabia, from August 2024 to June 2025. Al-Ahsa has a hot desert climate characterized by extremely hot summers (average daytime temperatures of 40–45 °C, occasionally exceeding 48 °C), mild winters (15–25 °C), low annual rainfall (<100 mm), and generally low relative humidity, except in irrigated areas [29]. Natural breeding was initiated in all does in October 2024. Pregnancies were confirmed by transabdominal ultrasonography using a Sonoscape E1 Expert ultrasound machine (Sonoscape Medical Corp., Shenzhen, Guangdong, China), and a total of 60 pregnant does were identified. These pregnant does were further selected for experiments based on the physiological stages of interest, such as late pregnancy and early lactation. During the course of the experiment, all does were kept under identical management conditions in shaded semi-open pens.

All animal experiments were performed in accordance with the ethical guidelines of the Institutional Animal Care and Use Research Ethics Committee (IACUC) of King Faisal University, Saudi Arabia. The experimental procedure was evaluated and approved by the committee (approval No. KFU-2026-ETHICS4364) on 10 May 2026.

### 2.2. Blood Sampling

Blood sampling was performed at three physiological stages during the study period. The first sampling was conducted at 8:00 a.m., when all examined does (n = 80) were non-pregnant and non-lactating; this stage was designated as Group 1 (non-pregnant, non-lactating). Following pregnancy diagnosis, 60 of the 80 enrolled does became pregnant and continued the longitudinal study. These 60 does were subsequently sampled at the remaining two physiological stages, whereas the 20 does that did not become pregnant contributed only baseline data and were excluded from the repeated-measures statistical analysis. A second blood sample was collected from the pregnant does (n = 60) during the final 2–4 weeks of gestation and designated as Group 2 (late pregnancy). The third blood sample was obtained from the same doe during the first 1–2 weeks postpartum and was designated Group 3 (early lactation).

Because the study followed the same animals longitudinally, blood sampling was performed according to the physiological stage rather than fixed calendar dates. Specifically, samples were collected before breeding (non-pregnant, non-lactating), during the last 2–4 weeks of gestation following ultrasonographic confirmation of pregnancy, and during the first two weeks postpartum. Therefore, the timing of sampling reflected each doe’s individual reproductive status, ensuring that all animals were evaluated at comparable physiological stages despite minor differences in conception and kidding dates.

The selection criteria were that all animals enrolled in the experiment should be clinically healthy, show normal estrus cycles, and have no reproductive pathologies. All pregnancies were confirmed by ultrasound, and all pregnant does were at the same stage of pregnancy during the second sampling (30 ± 5 days). All animals continued gestation normally and gave birth with no signs of dystocia or post-partum complications. All newborn kids were healthy and stayed with their mothers suckling milk until the weaning period at approximately three months of age.

Approximately 10 mL of blood was collected from the jugular vein of each doe and divided into two parts. One part of the blood was transferred into tubes with ethylenediaminetetraacetic acid (EDTA) for complete blood count (CBC) examination via an automated veterinary hematology analyzer (Exigo EOS Veterinary Hematology System, Boule Medical AB, Spånga, Stockholm, Sweden) and ribonucleic acid (RNA) isolation; another part was transferred into tubes without an anticoagulant for further serum extraction. Immediately after blood collection, all tubes were placed on crushed ice and delivered to the laboratory.

Whole blood was left to clot for 16 h at room temperature in plain tubes and then centrifuged at 3000 rpm for 15 min. Serum was subsequently harvested, fractionated into sterile microtubes, and stored at −20 °C for further testing of its biochemical, APP, metabolic, and oxidative stress profiles.

### 2.3. Biochemical Analysis

Serum biochemical, hormonal, acute-phase protein, and oxidative stress parameters were determined using commercially available assay kits, according to the manufacturers’ instructions.

Goat Serum Amyloid A ELISA Kit (IBL International GmbH, Hamburg, Germany); Goat Haptoglobin (HP) ELISA Kit (Eagle Biosciences Inc., Amherst, NH, USA); total protein, albumin, glucose, cholesterol, urea, creatinine and alkaline phosphatase (AP) diagnostic kits (Gamma Trade Co., Cairo, Egypt); Goat Insulin ELISA Kit (Neo-Biolab, Cambridge, MA, USA); triiodothyronine (T3), and thyroxine (T4) ELISA Kits (Bio-Diagnostic Co., Dokki, Giza, Egypt); malondialdehyde (MDA), Catalase (CAT), Reduced Glutathione (GSH), Superoxide Dismutase (SOD) and Glutathione Peroxidase (GPX) assay kits (Bio-Diagnostic Co., Dokki, Giza, Egypt); β-Hydroxybutyrate Assay Kit (Cayman Chemical, Ann Arbor, MI, USA); NEFA-HR (2) Assay Kit (FUJIFILM Wako Pure Chemical Corporation, Osaka, Japan); Goat insulin-like growth factor-1 ELISA Kit (Wuhan Fine Biotech Co., Ltd., Wuhan, Hubei, China); and Total Bilirubin Colorimetric Assay Kit (Atlas Medical GmbH, Dortmund, Germany).

### 2.4. Quantitative Real-Time Polymerase Chain Reaction (qPCR) Analysis

Total RNA was isolated from the whole blood of Shami goats at three physiologically distinct stages (non-pregnancy, late pregnancy, and early lactation) using TRI-zol Reagent (Invitrogen, Thermo Fisher Scientific, Waltham, MA, USA). Specifically, 250 µL of whole blood was mixed with 1 mL of TRIzol reagent and incubated at room temperature for 5 min to ensure complete denaturation of the nucleoprotein complex. Subsequently, 200 µL of chloroform was added, and the mixture was vortexed for 15 s, incubated for 3 min at room temperature, and centrifuged at 12,000× *g* for 15 min at 4 °C. Then, the aqueous layer comprising RNA was transferred into a new tube, and RNA was precipitated in 500 µL of isopropanol and centrifuged at 12,000× *g* for 10 min at 4 °C. The quantity and purity of RNA were evaluated using a NanoDrop ND-1000 spectrophotometer (Thermo Fisher Scientific, USA). First-strand complementary DNA (cDNA) was generated from the isolated RNA using the RevertAid First Strand cDNA Synthesis Kit (Thermo Fisher Scientific, Cat. No. EP0441) according to the manufacturer’s instructions.

Relative transcript abundance of genes involved in cellular energy metabolism; *sirtuin 1 (SIRT1)*, *peroxisome proliferator-activated receptor alpha (PPARA)*, *carnitine palmitoyltransferase 1A (CPT1A)*, *3-hydroxy-3-methylglutaryl-coenzyme A synthase 2 (HMGCS2)*, *cluster of differentiation 36 (CD36)*, *lipase E (LIPE)*, *protein kinase AMP-activated catalytic subunit alpha 1 (PRKAA1)*, *solute carrier family 2 member 1 (SLC2A1)*, *and solute carrier family 2 member 4 (SLC2A4)*, *oxidative stress and antioxidant defense (nuclear factor erythroid 2-related factor 2 (Nrf2)*, *heme oxygenase 1 (HMOX1)*, *superoxide dismutase 2 (SOD2)*, *glutathione peroxidase 1 (GPX1)*, *catalase (CAT)*, *and thioredoxin (TXN))*, *inflammatory and stress responses (haptoglobin (HP)*, *interleukin 6 (IL6)*, *and heat shock protein 70 (HSP70))*, *and autophagy (beclin 1 (BECN1) and autophagy-related 5 (ATG5))* was determined by quantitative real-time reverse transcription PCR (RT-qPCR) using 2× SensiFAST SYBR Lo-ROX Master Mix (Bioline, Cat. No. BIO-98002, London, UK). Gene-specific primers were designed using published caprine sequences deposited in the GenBank database, with the corresponding accession numbers listed in Table 1.

All amplification reactions were carried out in a total volume of 25 μL, including 12.5 μL of 2× SYBR Green Master Mix, 0.5 μL of forward and reverse primers each, 2 μL of cDNA template, and deionized water to make up to the desired reaction volume. The thermal cycling profile included an initial denaturing step at 95 °C for 10 min, 40 amplifying cycles (denaturation at 95 °C for 15 s, annealing at the optimum temperature for each primer set for 30 s, and extension at 72 °C for 30 s), and finally the analysis of a melting curve to check the specificity of each PCR product.

*GAPDH* was used as an internal control for normalizing gene expression. The relative abundance of messenger ribonucleic acid (mRNA) was quantified using the 2^−ΔΔCt^ method, with non-pregnant animals used as calibrators [30].

### 2.5. Statistical Analysis

Statistical analyses were conducted using the SPSS software package (version 20.0; IBM SPSS Statistics, Chicago, IL, USA). Descriptive statistics were calculated for all investigated variables, and the results are expressed as mean ± standard error (SE). Data normality was assessed using the Shapiro–Wilk test.

Because the same animals were evaluated repeatedly during three physiological stages (non-pregnancy, late pregnancy, and early lactation), the data were analyzed using a repeated-measures analysis of variance (repeated-measures ANOVA). In this model, the physiological stage was treated as a fixed within-subject factor, whereas animal identification (doe) was considered a repeated subject factor to account for the correlation among repeated measurements obtained from the same individual. Repeated-measures analyses were performed using data from 60 does that completed all three physiological stages. The 20 non-pregnant does that had no subsequent observations were excluded from the repeated-measures analysis.

No additional covariates or nuisance (noise) variables were included in the statistical model because all animals were clinically healthy Shami does of similar age (5–7 years), body weight (33–49 kg), management conditions, feeding regime, and housing, and each animal served as its own control across the three physiological stages. When a significant overall effect of the physiological stage was detected, pairwise comparisons among means were performed using Tukey’s honestly significant difference (HSD) post hoc test. Statistical significance was set at *p* < 0.05.

## 3. Results

### 3.1. Clinical Findings

Throughout the experimental period, all Shami does remained clinically healthy, with no evidence of systemic, metabolic, or reproductive disorders. All pregnancies progressed normally, and the does delivered healthy kids at term without obstetrical assistance. No cases of abortion, premature parturition, dystocia, retained fetal membranes, metritis, mastitis, or other postpartum complications were noted. Both dams and kids remained healthy throughout the study, and no neonatal morbidity or mortality was recorded during the pre-weaning period of the study.

### 3.2. Hematological Adaptations During Reproductive Transition

**Figure 1:**
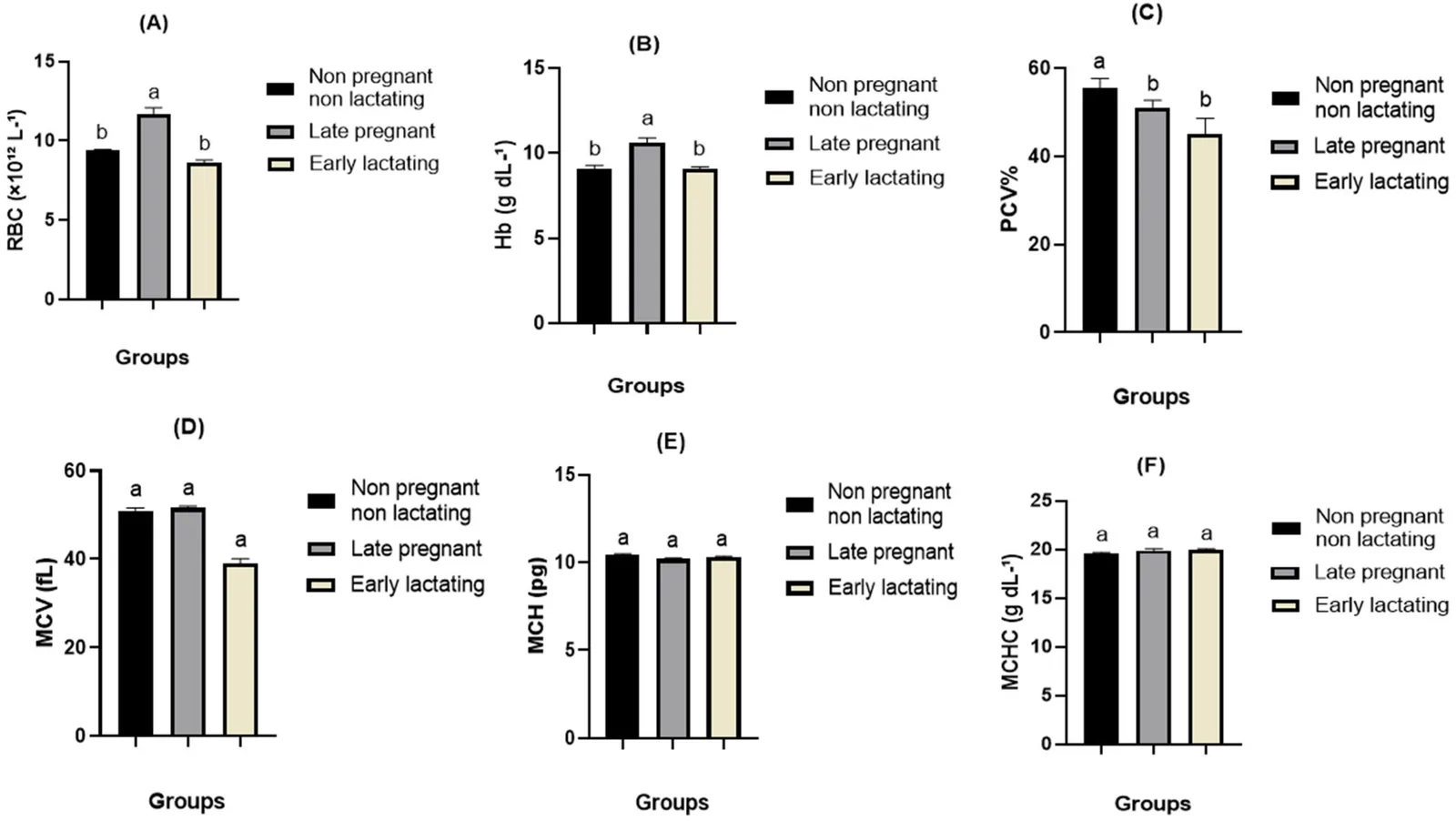
Hematological profiles of Shami goats during three physiological stages: non-pregnant non-lactating (control), late pregnancy, and early lactation. The evaluated erythrocytic parameters included (**A**) red blood cell count (RBC; ×10^12^ L^−1^), (**B**) hemoglobin concentration (Hb; g dL^−1^), (**C**) packed cell volume (PCV; %), (**D**) mean corpuscular volume (MCV; fL), (**E**) mean corpuscular hemoglobin (MCH; pg), and (**F**) mean corpuscular hemoglobin concentration (MCHC; g dL^−1^). Data are presented as mean ± standard error (SE). Bars with different lowercase letters (a, b) differ significantly among physiological stages (*p* < 0.05), whereas bars sharing the same letter indicate no significant difference.

**Figure 2:**
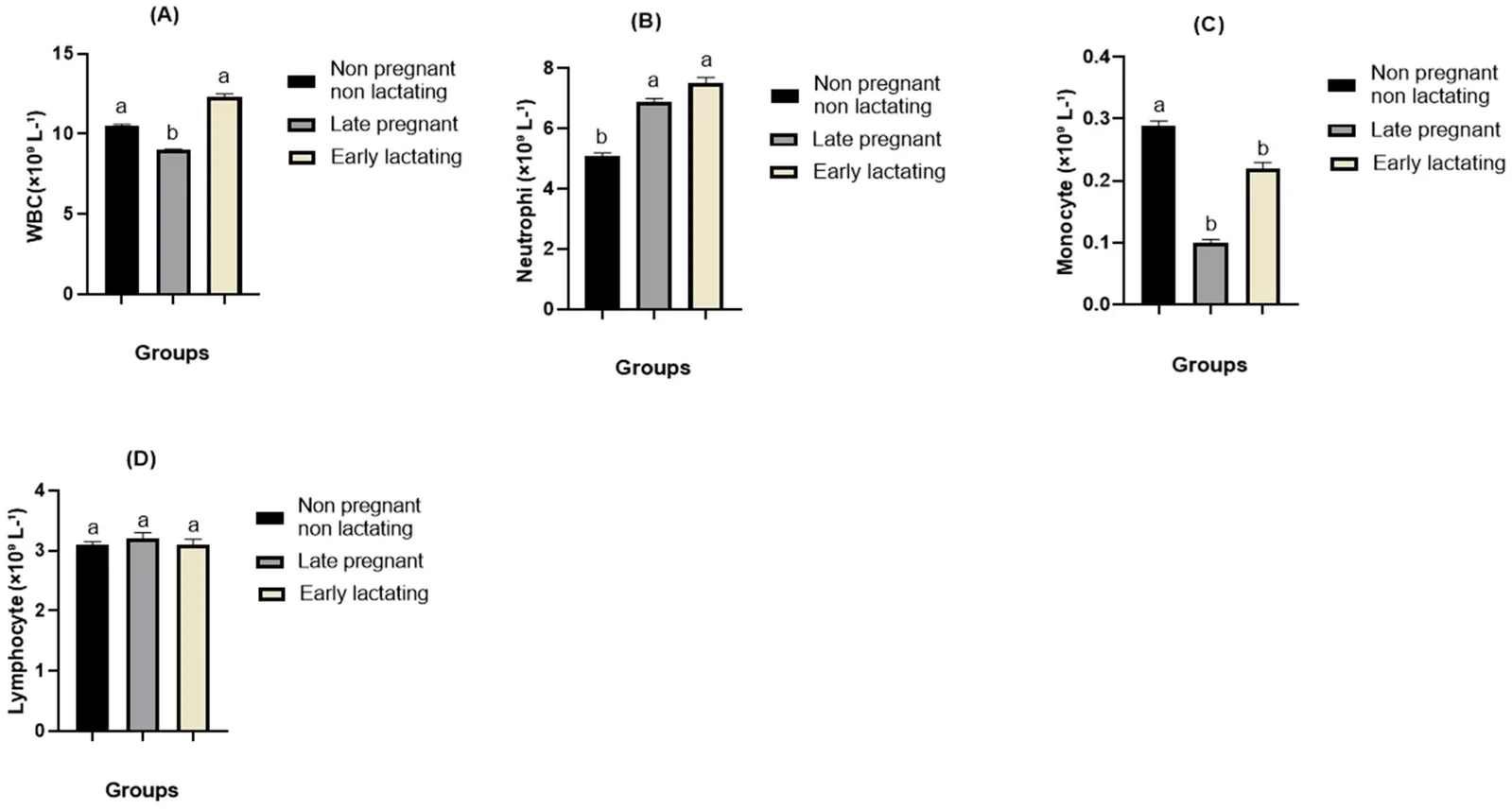
Leukocyte profiles of Shami goats during three physiological stages: non-pregnant non-lactating (control), late pregnancy, and early lactation. The leukocyte parameters included (**A**) total white blood cell count (WBC; ×10^9^ L^−1^), (**B**) neutrophil count (×10^9^ L^−1^), (**C**) monocyte count (×10^9^ L^−1^), and (**D**) lymphocyte count (×10^9^ L^−1^). Data are presented as mean ± standard error (SE). Bars with different lowercase letters (a–b) indicate statistically significant differences among physiological stages (*p* < 0.05), whereas bars sharing the same letter are not significantly different.

An overview of the hematological changes observed during reproductive transition is presented in Figure 1 and Figure 2. Significant stage-dependent alterations were observed in several hematological variables (*p* < 0.05). Late pregnancy was characterized by increased erythrocytic indices (red blood cell (RBC) count and hemoglobin (Hb)), accompanied by reduced packed cell volume (PCV) and total white blood cell (WBC) count, compared with the non-pregnant stage. Neutrophil percentages increased, whereas monocyte percentages decreased during late pregnancy and early lactation periods. The remaining hematological parameters were not significantly affected by the physiological stage.

### 3.3. Metabolic Adaptations During Reproductive Transition

The metabolic, hormonal, oxidative stress, and acute-phase protein profiles varied significantly among the physiological stages (Table 2 and Table 3). Late pregnancy and early lactation were associated with reduced circulating glucose, cholesterol, and antioxidant activities (GSH, GPx, SOD, and CAT), along with increased BHBA and haptoglobin concentrations (*p* < 0.05). Late pregnancy was characterized by increased albumin, globulin, urea, IGF-1, and MDA concentrations and reduced total protein and ALP activity. Early lactation exhibited the highest NEFA, T3, and T4 concentrations in the present study. In contrast, insulin, creatinine, total bilirubin, serum amyloid A, and fibrinogen levels did not differ significantly among the physiological stages.

### 3.4. Molecular Adaptations Associated with Reproductive Transition

**Figure 3:**
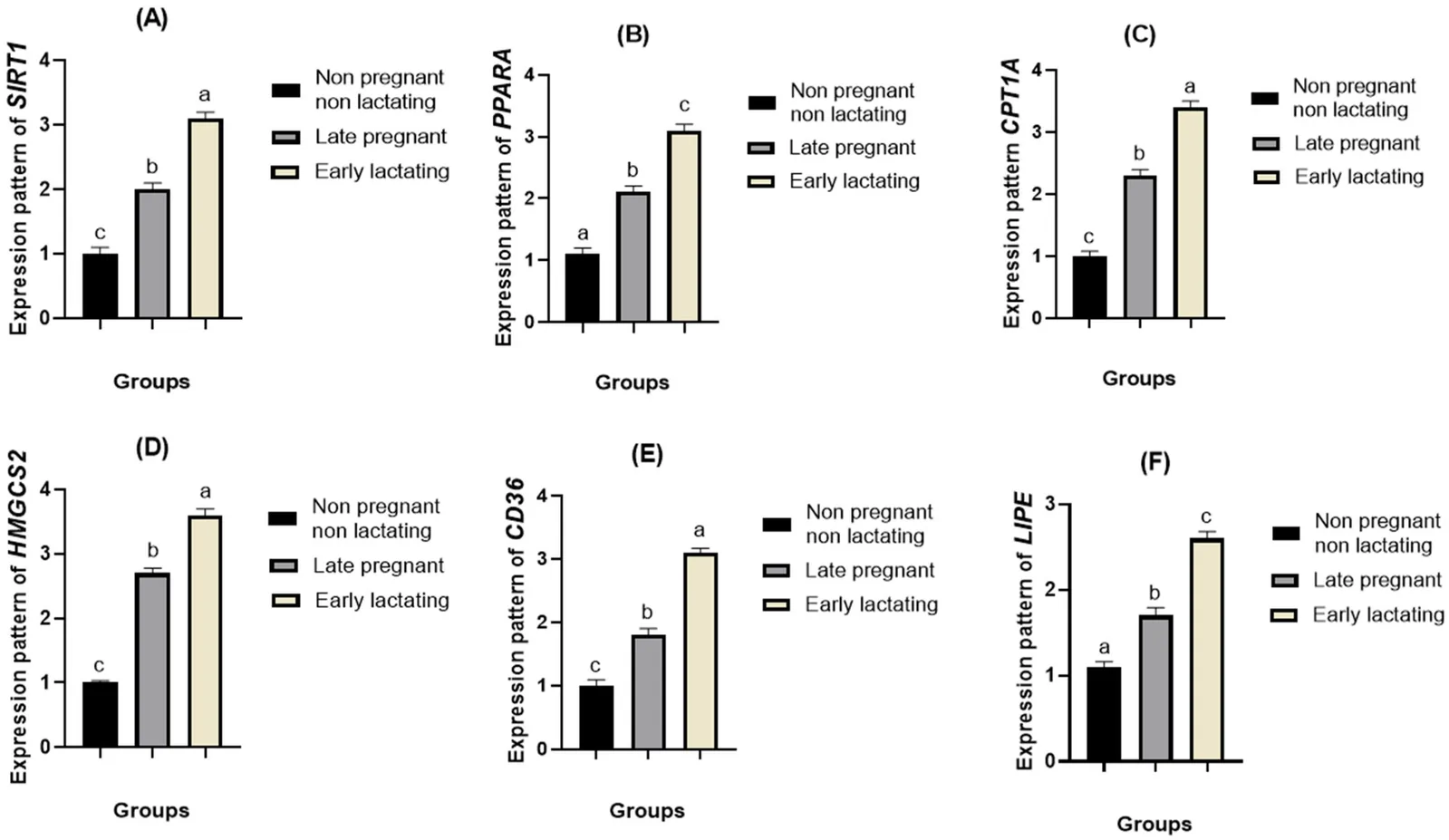

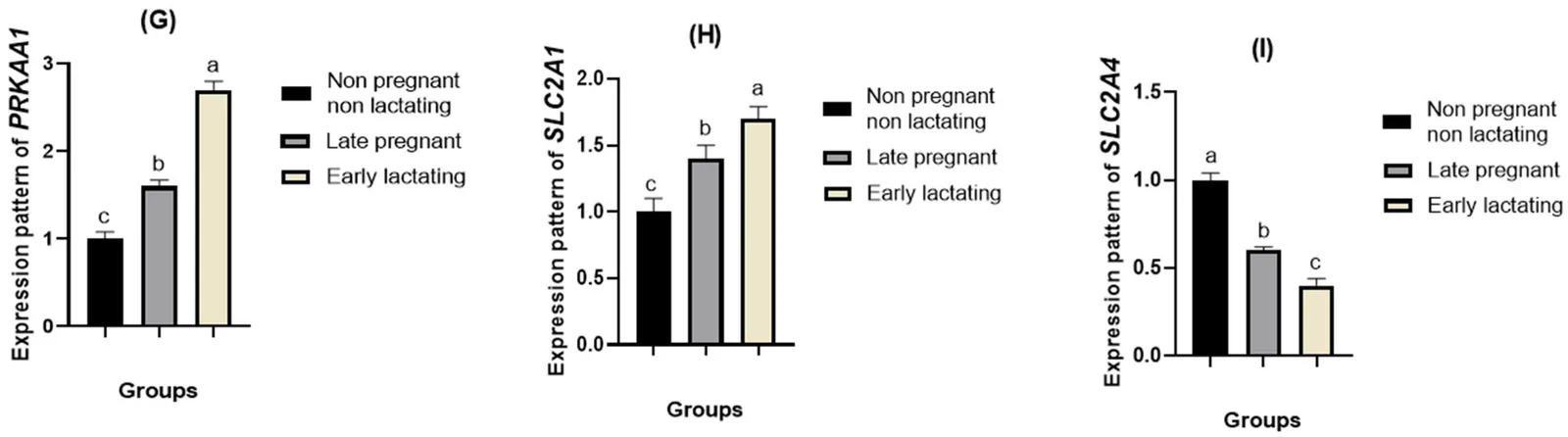
Relative mRNA expression of cellular energy sensing and lipid metabolism-related genes: *Sirtuin 1 (SIRT1)* (**A**), *Peroxisome proliferator-activated receptor alpha (PPARA)* (**B**), *Carnitine palmitoyltransferase 1A (CPT1A)* (**C**), *3-Hydroxy-3-methylglutaryl-CoA synthase 2 (HMGCS2)* (**D**), *Cluster of differentiation 36 (CD36)* (**E**), *Hormone-sensitive lipase (LIPE)* (**F**), *Protein kinase AMP-activated catalytic subunit alpha 1 (PRKAA1)* (**G**), *Solute carrier family 2 member 1 (SLC2A1)* (**H**), and *Solute carrier family 2 member 4 (SLC2A4)* (**I**) in Shami goats during the pre-pregnancy, late pregnancy, and early lactation stages. Data are presented as mean ± SEM. Different superscript letters (a, b, c) within the same gene indicate significant differences among physiological stages (*p* < 0.05).

**Figure 4:**
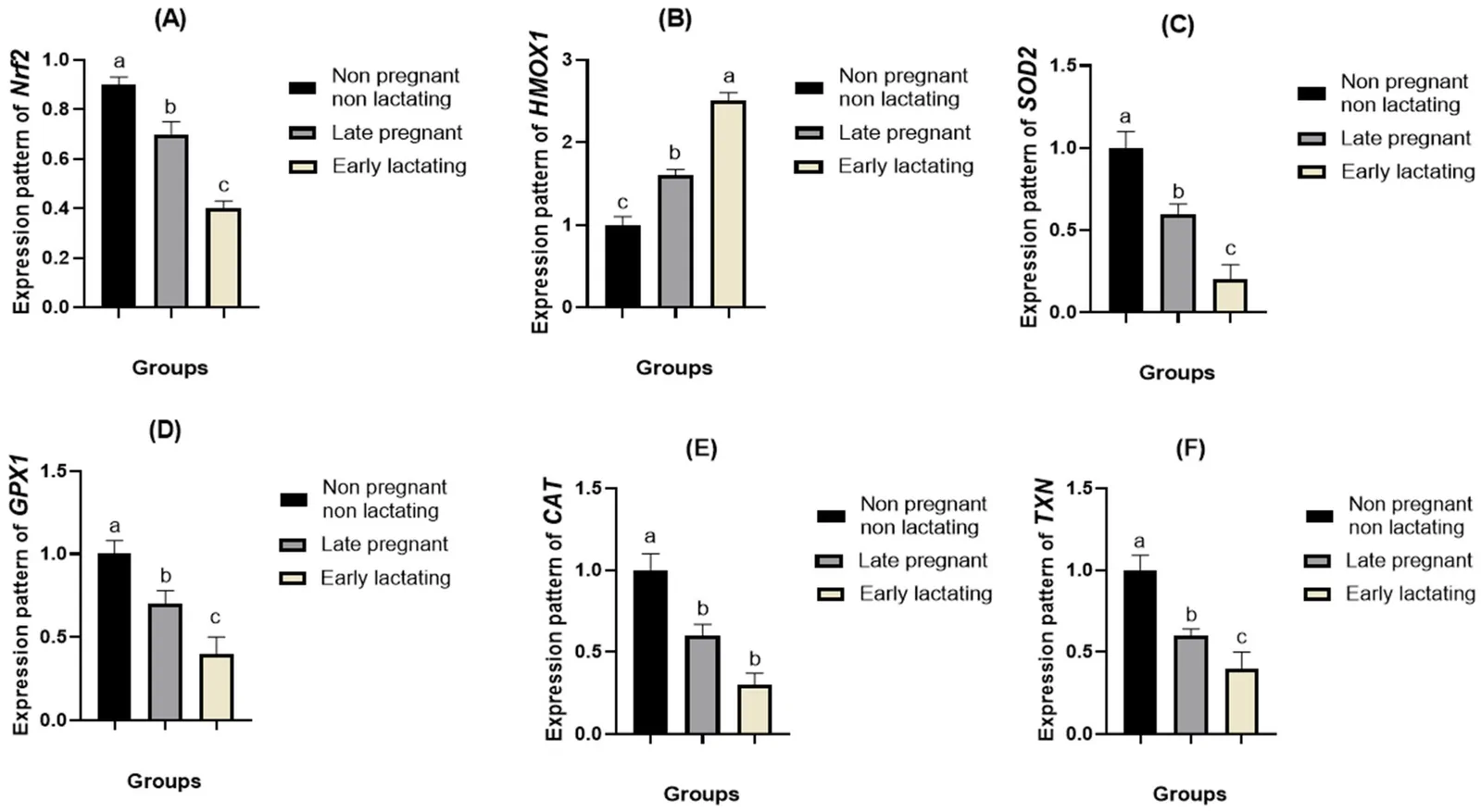
Relative mRNA expression of oxidative stress and antioxidant defense-related genes: *Nuclear factor erythroid 2-related factor 2 (Nrf2)* (**A**), *Heme oxygenase 1 (HMOX1)* (**B**), *Superoxide dismutase 2 (SOD2)* (**C**), *Glutathione peroxidase 1 (GPX1)* (**D**), *Catalase (CAT)* (**E**), and *Thioredoxin (TXN)* (**F**) in Shami goats during the pre-pregnancy, late pregnancy, and early lactation stages. Data are presented as mean ± SEM. Different superscript letters (a, b, c) within the same gene indicate significant differences among physiological stages (*p* < 0.05).

**Figure 5:**
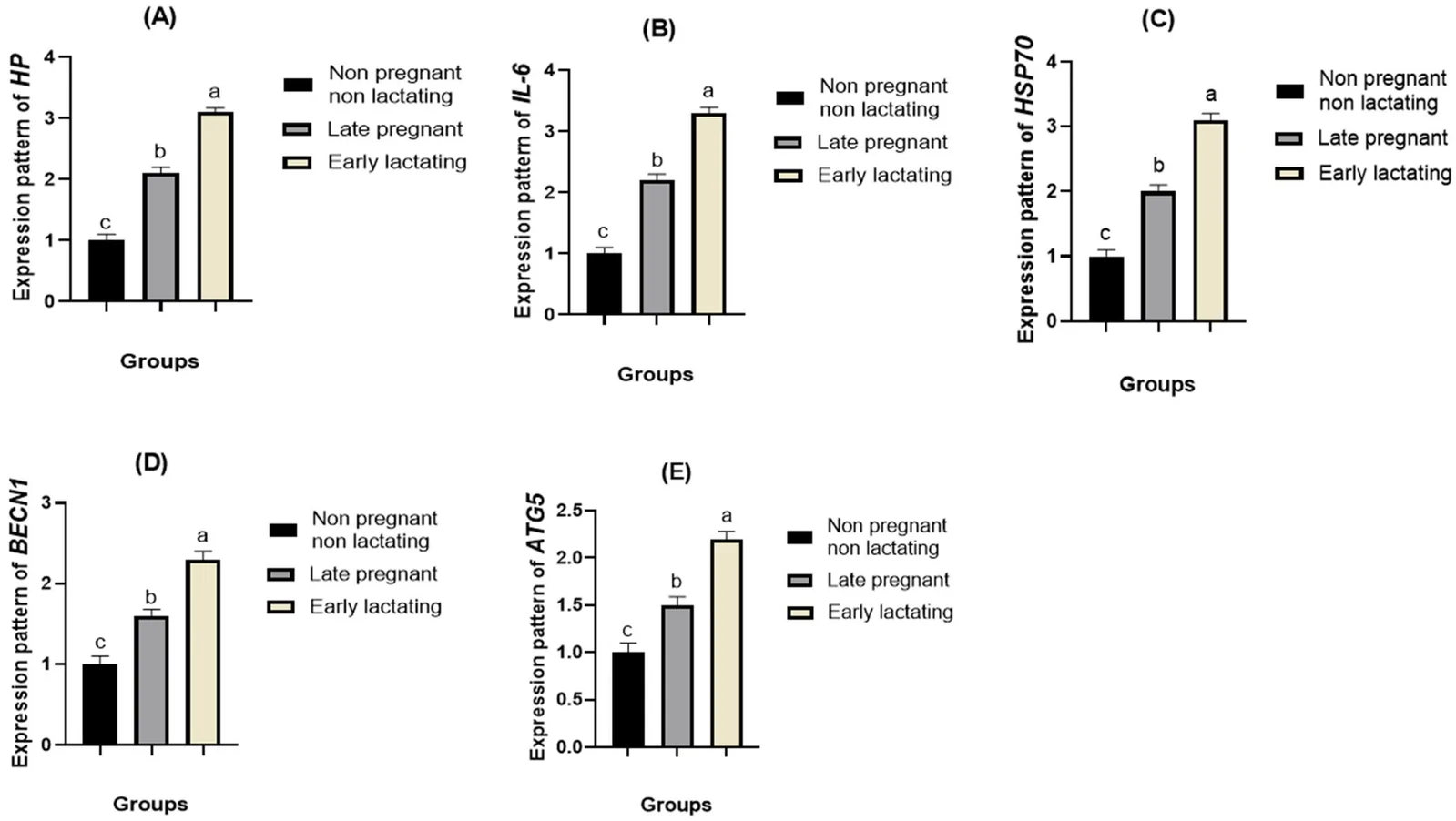
Relative mRNA expression of inflammatory response, cellular stress response, and autophagy-related genes: *Haptoglobin (HP)* (**A**), *Interleukin 6 (IL6)* (**B**), *Heat shock protein 70 (HSP70)* (**C**), *Beclin 1 (BECN1)* (**D**), and *Autophagy-related 5 (ATG5)* (**E**) in Shami goats during the pre-pregnancy, late pregnancy, and early lactation stages. Data are presented as mean ± SEM. Different superscript letters (a, b, c) within the same gene indicate significant differences among physiological stages (*p* < 0.05).

Distinct stage-dependent transcriptional changes were observed in genes associated with cellular energy sensing and lipid metabolism (*SIRT1*, *PPARA*, *CPT1A*, *HMGCS2*, *CD36*, *LIPE*, *PRKAA1*, and *SLC2A1*), oxidative stress (*Nrf2*, *GPX1*, *CAT*, *SOD2*, *TXN*, and *SLC2A4*), inflammation, and autophagy (*HMOX1*, *HP*, *IL6*, *HSP70*, *BECN1* and *ATG5*) (Figure 3, Figure 4 and Figure 5). Genes involved in energy sensing, lipid metabolism, inflammatory responses, cellular stress, and autophagy generally exhibited progressive upregulation from the non-pregnant stage through late pregnancy, reaching their highest expression levels during early lactation (*p* < 0.05). Conversely, genes related to antioxidant defense and glucose transport showed the opposite pattern, with highest expression in non-pregnant goats and a progressive decline during late pregnancy and early lactation (*p* < 0.05).

## 4. Discussion

The present study was designed to characterize the hematological, biochemical, hormonal, oxidative stress, and molecular adaptations associated with the transition from the non-pregnant state through late pregnancy to early lactation in Shami goats. The findings demonstrated that this physiological transition was accompanied by significant changes in hematological indices, energy metabolism, endocrine status, oxidative stress biomarkers, and expression of genes involved in lipid metabolism, antioxidant defense, inflammation, and autophagy. Collectively, these results achieved the study objective by providing a comprehensive characterization of the physiological and molecular adaptations that occur during reproductive transition in Shami goats and identifying potential biomarkers associated with these critical physiological stages.

The hematological study showed significantly higher levels of RBC and Hb concentrations during late pregnancy compared to those during the pre-pregnancy and early lactation periods; however, PCV significantly decreased during late pregnancy and early lactation (Figure 1). Similar results have been observed in sheep [31] and goats [32], but no significant changes have been found in cattle [33]. The high RBC and Hb levels may be due to high oxygen consumption, while the low PCV may occur because of dilution of blood due to an increase in plasma volume during pregnancy and early lactation [34].

Total number of leukocytes was significantly lower during late pregnancy compared to pre-pregnancy and early lactation (Figure 2), which is in agreement with Ossimi sheep [35]. However, a higher number of leukocytes during lactation has been reported in buffaloes [36], but an increased number of leukocytes during pregnancy has been shown in sheep [37]. The reduced leukocyte count during pregnancy may be related to physiological adaptations associated with gestation, while the increase during early lactation may reflect postpartum uterine involution and recovery [38].

The differential leukocyte count demonstrated significant neutrophilia accompanied by reduced monocyte counts during late pregnancy and early lactation (Figure 2), in agreement with previous reports in cattle and Holstein cattle [33,36]. Lymphocyte counts remained relatively unchanged, consistent with observations in cross-bred goats [39]. These changes are likely mediated by pregnancy-associated stress, which stimulates adrenocorticotropic hormone (ACTH) secretion and glucocorticoid release, promoting neutrophil mobilization into the peripheral circulation [40].

Compared to the pre-conception period, Shami goats had significantly reduced glucose levels during late gestation and early lactation and reduced TP levels during late gestation, as presented in Table 2. Similar results have been previously reported in sheep [17] and dairy cows [41,42]. In contrast, elevated glucose and TP levels were recorded during early lactation in dromedary camels [43], whereas no change in TP levels was observed in dairy cattle [33]. The decreased glucose levels may be due to the increased utilization of glucose by the fetus and the increased energy requirements for milk production [44,45]. Similarly, decreased TP levels may be attributed to the increased protein requirements of fetal development and colostrum production [46,47].

Serum cholesterol levels were also significantly lower in late-pregnant and early lactating Shami goats (Table 2), which is consistent with similar studies on goats [48,49]. However, increased cholesterol levels have been reported in Carpathian Romanian buffaloes [50] and crossbred goats [39], while no differences have been reported between different physiological periods in West African dwarf does [51]. The reduction in cholesterol in this study is probably due to enhanced cholesterol use for steroid synthesis and mammary gland activity.

Serum albumin and globulin levels were significantly higher in late pregnancy than in early lactation in Shami goats (Table 2). These results coincide with earlier data in sheep [17] and do not match reports in camels [52] and dairy cows [53]. The high albumin level in late pregnant goats can be explained by the higher nutritional and metabolic needs required to support fetal development [54]. The reduction in albumin in early lactation is related to the increased transfer of plasma proteins, particularly immunoglobulins, into colostrum [55]. The same explanation applies to the high globulin levels in goats during late pregnancy [55,56].

As shown in Table 2, serum urea concentrations were considerably raised during late pregnancy, in agreement with findings in sheep [35] and dairy cows [22], although no significant changes have been reported in dairy cows [52]. The increase in urea likely reflects enhanced protein turnover and amino acid catabolism to meet the increased metabolic demands of pregnancy [45]. In contrast, serum creatinine and total bilirubin concentrations remained unchanged throughout the studied physiological stages, consistent with previous reports in sheep [34], cross-bred goats [39], and West African dwarf does [51], but differing from [24], who reported increased creatinine concentrations during late pregnancy. Serum ALP activity was significantly higher during the pre-pregnancy stage, consistent with observations in dairy cows [53] but contrasting with [57], who reported higher ALP activity in early-lactation dairy cows. Variations in ALP activity among physiological stages may reflect changes in hepatic metabolism and feed intake around parturition, together with the metabolic stresses related to fetal development and the start of lactation [58].

It is evident from Table 2 that the serum BHBA concentrations were higher at late pregnancy and early lactation than those recorded at the pre-pregnancy phase, similar to the results observed in sheep [59]; however, Ref. [33] showed that the highest BHBA concentrations were observed in non-pregnant cattle. Higher BHBA concentrations result from higher energy needs and greater fat mobilization due to a negative energy balance during the transition period.

The highest serum NEFA concentrations were recorded during early lactation, similar to the results in cattle [33]; however, unlike [60], we did not observe any significant differences in sheep. The increase in serum NEFA concentrations is a result of increased lipolysis in adipose tissue to supply energy for milk production. Higher concentrations of growth hormones and lower concentrations of insulin in early lactation contribute to greater lipid mobilization [60].

Serum T3 and T4 concentrations were significantly higher during early lactation than during the other physiological stages (Table 2). These findings agree with previous studies in sheep [61] and cattle [33,41,62], although increased thyroid activity during pregnancy has also been reported in Carpathian Romanian buffaloes [50] and goats [63]. The elevated thyroid hormone concentrations during early lactation likely reflect their essential role in regulating energy metabolism and supporting milk synthesis [54]. IGF-1 concentrations were highest during late pregnancy, consistent with observations in Gray Shirazi ewes [59] but differing from those reported by [37] in sheep. The increased IGF-1 concentration during late gestation likely reflects its critical role in fetal growth, placental function, and maternal metabolic adaptation [64].

In the current study, there was a notable increase in MDA content, along with a substantial decrease in GSH, GPx, CAT, and SOD levels, at the late pregnancy and early lactation stages compared to the pre-pregnancy phase (Table 3). These results are in accordance with those of other studies performed on native and crossbred cattle [65], camels [24,66], and West African dwarf does [51]. The observed oxidative stress status is attributed to the enhanced formation of ROS due to the presence of metabolic demand, negative energy balance, and fatty acid and protein catabolism during the late gestational and early lactation phases.

Among the acute-phase proteins evaluated, only Hp showed a significant increase during early lactation (Table 3), which agrees with previous findings in dairy cows [21,67] and goats [19]. This increase is likely connected to the negative energy balance commonly observed in dairy cattle during early lactation, when energy requirements for milk production exceed dietary energy intake, stimulating the acute-phase response [68].

In contrast, SAA and Fb concentrations did not differ significantly among the physiological stages, consistent with the observations in Saanen goats [19]. However, other studies have reported significant changes in SAA levels throughout pregnancy, lactation, and parturition [21,67,69,70]. These inconsistencies may be explained by differences in breed, nutrition, management practices, season, age, parity, reproductive status, and milk production, all of which can influence the acute-phase protein responses.

In the current study, genes linked to energy metabolism, including *SIRT1*, *PPARA*, *CPT1A*, *HMGCS2*, *CD36*, *LIPE*, *PRKAA1*, and *SLC2A1*, as well as *HMOX1*, *HP*, *IL6*, *HSP70*, *BECN1*, and *ATG5*, exhibited a progressive increase in expression from the non-pregnant stage through late pregnancy to early lactation. In contrast, the antioxidant defense genes *NFE2L2*, *GPX1*, *CAT*, *SOD2*, and *TXN*, together with the glucose transporter gene *SLC2A4*, showed a progressive decline in expression across the same physiological stages (Figure 3, Figure 4 and Figure 5). A coordinated upregulation of *SIRT1*, *PRKAA1*, *PPARA*, *CPT1A*, *HMGCS2*, *CD36*, *LIPE*, and *SLC2A1*, together with downregulation of *SLC2A4*, indicated a tightly regulated metabolic shift toward lipid utilization and glucose sparing.

The progressive increase in *SIRT1* and *PRKAA1* suggests activation of the *AMPK–SIRT1* signaling network, a conserved energy-sensing axis that orchestrates metabolic adaptation to nutrient limitation. *AMPK* activation enhances catabolic pathways while inhibiting adenosine triphosphate (ATP)-consuming processes, whereas *SIRT1* promotes mitochondrial biogenesis and fatty acid oxidation via deacetylation of peroxisome proliferator-activated receptor gamma coactivator 1-α and related transcription factors [71,72]. Similar activation of *AMPK* and *SIRT1* has been reported in transition dairy cows [73], energy-restricted goats [74], and high-yielding buffaloes [74], supporting their conserved roles in ruminant metabolic adaptation.

PPARA upregulation represents a higher regulation of lipid catabolic processes at the transcriptional level. Indeed, PPARα is potently stimulated by NEFA and controls the expression of genes controlling mitochondrial β-oxidation. Supporting this, elevated hepatic PPARA expression has been found in dairy cows during early lactation [75], goats under feed deprivation [76], and sheep undergoing NEB. Higher induction of CPT1A further proves increased fatty acid transport to the mitochondria and their oxidation. CPT1A is a well-known metabolic bottleneck in the β-oxidation pathway, and its induction during the transition period has been described in cattle [77] and goats [78].

High expression of HMGCS2 shows increased ketone body production, as seen by the high concentrations of BHBA in this experiment. HMGCS2 is the rate-limiting enzyme of ketogenesis and is induced when acetyl-coenzyme A (CoA) concentrations are higher than the capabilities of the tricarboxylic acid cycle. Such induction was found in dairy cows predisposed to ketosis [79], goats under metabolic stress, and buffaloes during maximal lactation.

Increased expression of *LIPE* and *CD36* reflects intensified adipose tissue lipolysis and fatty acid uptake in the liver. Hormone-sensitive lipase (LIPE) catalyzes triglyceride breakdown, and *CD36* facilitates cellular fatty acid transport. These outcomes are consistent with those of previous studies in dairy cows [80], goats under NEB, and sheep in late gestation, where enhanced lipid mobilization supports energy demands.

The opposing regulation of glucose transporter upregulation of *SLC2A1* and downregulation of *SLC2A4* reflects adaptive glucose redistribution. *GLUT1* supports basal glucose uptake, whereas reduced glucose transporter 4 (GLUT4) limits insulin-dependent glucose utilization in peripheral tissues, ensuring glucose availability for lactose synthesis in the mammary gland. Similar *GLUT4* suppression has been reported in dairy cows [81], goats, and sheep during early lactation and is considered a hallmark of physiological insulin resistance in ruminants.

Energy metabolism-related genes (*SIRT1*, *PPARA*, *CPT1A*, *HMGCS2*, *CD36*, *LIPE*, *PRKAA1*, and *SLC2A1*), along with *HMOX1*, *HP*, *IL6*, *HSP70*, *BECN1*, and *ATG5*, were gradually upregulated in the current study from the non-pregnant stage to early lactation, while antioxidant defense genes (*Nrf2*, *GPX1*, *CAT*, *SOD2*, and *TXN*) and the glucose transporter gene *SLC2A4*) were gradually downregulated. These alterations indicate a concerted defense against oxidative and metabolic stressors, including autophagy, inflammatory signaling, antioxidant defense, and cellular stress adaptation. Transition dairy cattle and other ruminants under metabolic stress have been shown to have comparable molecular reactions [82].

Oxidative stress during the periparturient period arises primarily from increased mitochondrial activity associated with enhanced β-oxidation and ketogenesis, which leads to excessive ROS production. Similar oxidative stress patterns have been widely reported in dairy cows [57], goats [83], sheep, and buffaloes, particularly during the early lactation period. Supporting these findings, El-Sayed et al. [84] reported significant stage-dependent modulation of antioxidant defense and cytokine gene expression across physiological stages, with the strongest oxidative and inflammatory activation occurring during late pregnancy and early lactation. Their study demonstrated that antioxidant genes and redox regulators closely follow metabolic stress intensity, confirming that periparturient oxidative stress is a conserved response in small ruminants.

Similarly, in dromedary camels, Ateya et al. [43] showed that serum antioxidant markers and their gene expression profiles could clearly discriminate the periparturient period, with marked activation of antioxidant defense systems around parturition. These findings strongly support the present results and reinforce the notion that activation of the NRF2–antioxidant axis is a universal adaptive mechanism in large and small ruminants during metabolic transition.

The upregulation of *NRF2* suggests the activation of the master antioxidant regulatory pathway. *NRF2* controls the transcription of key detoxifying enzymes, including *HMOX1*, *SOD2*, *CAT*, *GPX1*, and *TXN*, via antioxidant response elements. In dairy cattle, *NRF2* activation has been linked to improved oxidative resilience during early lactation [85], while similar reactions have been described in goats exposed to heat and metabolic stress and buffaloes under high production pressure. Increased *HMOX1* expression reflects enhanced haem degradation and antioxidant bilirubin production. *HMOX1* is a sensitive biomarker of oxidative stress and has been consistently elevated in cows, goats, and sheep exposed to metabolic overload or inflammation [83,86].

The coordinated upregulation of *SOD2*, *CAT*, *GPX1*, and *TXN* indicates the activation of both mitochondrial and cytosolic antioxidant systems. *SOD2* detoxifies superoxide radicals, whereas *CAT* and *GPX1* eliminate hydrogen peroxide, maintaining redox equilibrium. *TXN* further supports thiol-based redox regulation. Similar antioxidant enzyme activation has been reported in dairy cows [85], goats, sheep, and buffaloes during the transition and heat stress conditions.

The increased expression of *HSP70* reflects the activation of molecular chaperone systems that prevent protein misfolding and aggregation under stress conditions. *HSP70* is a well-documented biomarker of metabolic stress in dairy cows [87], goats, and buffaloes, particularly during early lactation when metabolic demand peaks.

The upregulation of IL-6 and HP indicates the activation of inflammatory and acute-phase responses. IL-6 is a pivotal cytokine that links immune activation to metabolic regulation by stimulating hepatic acute-phase protein synthesis and coordinating inflammatory signaling [88,89]. Haptoglobin is an important positive acute-phase protein whose levels rise as a result of oxidative stress, trauma, and inflammation in animals, thus acting as a marker of metabolic stress in ruminants [90]. Higher levels of IL-6 and haptoglobin have been found in dairy cattle undergoing negative energy balance (NEB) and metabolic stress, and such changes have also been noted in sheep, goats, and buffaloes at the time of parturition [88,89,90].

In dairy cows, RNA extracted from peripheral blood and analyzed using real-time PCR revealed the dynamic regulation of Toll-like receptors (*TLR2*, *TLR4*, *TLR6*, and *TLR7*) and *β-defensin 5 (BNBD5)* across the transition period. Specifically, pro-inflammatory immune receptors, such as *TLR4*, *TLR6*, *TLR7*, and *BNBD5*, were significantly upregulated in the week postpartum, whereas earlier stages exhibited comparatively lower expression levels. In contrast, *TLR2*, a receptor often associated with immunomodulatory and tolerance-related signaling, was transiently upregulated at calving and during the first week postpartum, before declining thereafter [36].

Increased expression of *BECN1* and *ATG5* indicates autophagy activation, a key adaptive mechanism for recycling damaged cellular components and maintaining energy homeostasis under metabolic stress. Autophagy is increasingly recognized as an essential survival pathway during the transition period in dairy ruminants, particularly under conditions of NEB, where it contributes to cellular quality control and metabolic flexibility [91,92,93].

The current study has several limitations. First, the research was performed in only one herd during one production year, involving a relatively small number of Shami goats. Second, owing to the cross-sectional design, the comparison of the physiological status of the groups was made at one point in time and not longitudinally; thus, it was impossible to assess the changes within the same animals over time. Third, the research was performed using only one goat breed; thus, the results cannot be generalized to other breeds with different genotypes. Fourth, although a wide range of hematological, biochemical, hormonal, inflammatory, oxidative stress, and metabolic biomarkers were investigated, molecular analysis was performed for only some immune- and antioxidative-related genes. Finally, the current investigation was based solely on physiological and molecular biomarkers, while functional or phenotypic indicators, such as body condition score, milk yield, reproduction performance, or productivity traits, were not studied.

Considering all these factors, the combination of the parameters mentioned above should be included in future investigations to provide a more comprehensive assessment of the biological meaning and practical value of physiological adaptations.

## 5. Conclusions

Understanding the physiological and molecular adaptations that occur during the transition period is essential for improving the health, welfare, reproductive performance, and productivity of dairy goats. The present study demonstrated that the transition from late pregnancy to early lactation in Shami goats is accompanied by coordinated physiological and molecular adaptations involving energy metabolism, lipid mobilization, antioxidant defense, inflammatory responses, and cellular homeostasis. Gene expression analyses indicated the activation of energy-sensing pathways, fatty acid oxidation, inflammatory responses, and autophagy, along with changes in antioxidant defense mechanisms, reflecting the increased metabolic demands associated with the transition period. These molecular alterations were accompanied by significant hematological, biochemical, hormonal, oxidative stress, and inflammatory changes, with early lactation being the most physiologically demanding stage. Collectively, these findings indicate that successful reproductive transition in Shami goats depends on integrated physiological, metabolic, immunological, and molecular adaptations. The identified biomarkers may provide valuable tools for monitoring maternal adaptation and reproductive status during the periparturient period and may contribute to the development of management strategies aimed at improving the health and productivity of animals.

Future research should validate these findings in larger populations, multiple goat breeds, and diverse production systems to improve generalizability. In addition, integrating transcriptomic, proteomic, and metabolomic approaches, together with protein-level validation and functional studies, would provide a more comprehensive understanding of the molecular mechanisms regulating the transition period and further confirm the diagnostic and prognostic values of the identified biomarkers.

## Figures and Tables

**Table 1:** Real-time polymerase chain reaction (RT-PCR) primers made of oligonucleotides that are forward and reverse for genes under study.

Investigated Marker	Primer	Product Size (bp)	Annealing Temperature (°C)	GenBank Isolate
*SIRT1*	F5′-GGCTTACAGGGCCTATCCAG-3′ R5′-ACACGAATGGAAACCGTTGG-3′	156	55	NM_001314319.1
*PPARA*	F5′-CACAAGTGCCTTTCCGTTGG-3′ R5′-ATGACGAAGGGCGGATTGTT-3′	248	58	HM600811.1
*CPT1A*	F5′-TGAGTGACTGGTGGGAGGAA-3′ R5′-CAGAGCGGAATCGTAGACCC-3′	217	58	XM_018043311.1
*HMGCS2*	F5′-TGCCTCCCTCTTCAATGCTG-3′ R5′-TAAGCCCTCTCTCGAGGACC-3′	182	60	GU586190.1
*CD36*	F5′-GGCAACTGTATCTTTCTATTGTGA-3′ R5′-AGCATGTCTCCAACTGGCAT-3′	214	55	OQ397575.1
*LIPE*	F5′-TCACCGAGATCCAGGTGCTA-3′ R5′-GATAAGCCTGACGAGGACGG-3′	185	58	GQ927175.1
*PRKAA1*	F5′-TGGTTGCTGAAACTCCCAGG-3′ R5′-TCTTCCTCCGAACACGCAAA-3′	213	60	PP417847.1
*SLC2A1*	F5′-GAGATGCTGATCCTGGGTCG-3′ R5′-CTGGTTGCCCATGATGGAGT-3′	192	60	NM_001314223.1
*SLC2A4*	F5′-CAGCTGCCTCCTACGAGATG-3′ R5′-CTAGCACCTGGGCGATTAGG-3′	180	58	NM_001314227.1
*Nrf2*	F5′-CTGTTCTCTGCTGTCAAGGGA-3′ R5′-ACTCGCCGGTCTCTTCATCT-3′	221	58	NM_001314327.1
*HMOX1*	F5′-CAAGCGCTATGTTCAGCGAC-3′ R5′-GCTTGAACTTGGTGGCACTG-3′	206	55	NM_001285567.1
*SOD2*	F5′-GCTTGCAGATTGCTGCTTGT-3′ R5′-TGGCCTTCAGATAATCGGGC-3′	138	60	XM_018053428.1
*GPX*	F5′-TGTGGTTTACGGATCCTGGC-3′ R5′-CCCTTGGGCTGGACTTTCAT-3′	182	58	NM_001285712.2
*CAT*	F5′-GAGGAAACGCCTGTGTGAGA-3′ R5′-GGATGCGGGAGCCATATTCA-3′	182	58	GQ204786.1
*TXN*	F5′-GCCTTGCATCCGTTTCCATC-3′ R5′-ACCATGTGGCTGAGAAGTCG-3′	156	58	XM_005684290.3
*HP*	F5′-TAACCTCATCTCGGGAGCCA-3′ R5′-ACCATGTGGCTGAGAAGTCG-3′	172	60	PP328486.1
*IL-6*	F5′-TTCAGTCCACTCGCTGTCTC-3′ R5′-TGCTTGGGGTGGTGTCATTC-3′	106	58	NM_001285640.1
*HSP70*	F5′-GGGGAGGACTTCGACAACAG-3′ R5′-GGCTGATGTCCTTCTTGTGCT-3′	76	60	JF412690.1
*BECN1*	F5′-AGCCTCTGAAACTGGACACG-3′ R5′-GGGGGATGAATCTGCGAGAG-3′	183	58	XM_005693865.3
*ATG5*	F5′-GAGCATGTCACCCTTCTGCT-3′ R5′-TCATGTCGCAGCTGAGGTTT-3′	175	60	XM_018053114.1
*GAPDH*	F5′-ATCAAGTGGGGTGATGCTGG-3′ R5′-TACTTCTCGTGGTTCACGCC-3′	167	60	AJ431207.1

*SIRT1*, *Sirtuin 1*; *HMOX1*, *Heme oxygenase 1*; *SOD2*, *Superoxide dismutase 2 (mitochondrial)*; *PPARA*, *Peroxisome proliferator-activated receptor alpha*; *CPT1A*, *Carnitine palmitoyltransferase 1A*; *HMGCS2*, *3-Hydroxy-3-methylglutaryl-CoA synthase 2*; *CD36*, *Cluster of differentiation 36 (fatty acid translocase)*; *LIPE*, *Lipase E (hormone-sensitive lipase)*; *PRKAA1*, *Protein kinase AMP-activated catalytic subunit alpha 1*; *SLC2A1*, *Solute carrier family 2 member 1*; *SLC2A4*, *Solute carrier family 2 member 4*; *Nrf2*, *Nuclear factor erythroid 2-related factor 2*; *GPX1*, *Glutathione peroxidase 1*; *CAT*, *Catalase*; *TXN*, *Thioredoxin*; *HSP7*, *Heat shock protein 70*; *HP*, *Haptoglobin*; *IL6*, *Interleukin 6*; *BECN1*, *Beclin 1*; *ATG5*, *Autophagy-related 5*; *GAPDH*, *Glyceraldehyde-3-phosphate dehydrogenase*.

**Table 2:** Biochemical and hormonal parameters in Shami goats during different physiological states (mean ± SE).

Parameters	Non Pregnant	Late Pregnant	Early Lactating	*p*-Value
Glucose (mmol L^−1^)	5.13 ± 0.07 ^a^	4.17 ± 0.06 ^b^	4.03 ± 0.02 ^b^	0.001
Cholesterol (mmol L^−1^)	2.10 ± 0.01 ^a^	1.28 ± 0.01 ^b^	1.30 ± 0.01 ^b^	0.001
Total protein (g L^−1^)	68.10 ± 1.01 ^a^	43.12 ± 0.81 ^b^	68.11 ± 0.50 ^a^	0.001
Albumin (g L^−1^)	33.10 ± 1.02 ^b^	49.11 ± 0.51 ^a^	34.13 ± 1.01 ^b^	0.001
Globulin (g L^−1^)	140.10 ± 0.50 ^b^	26.10 ± 1.10 ^a^	15.10 ± 0.51 ^b^	0.001
Urea (mmol L^−1^)	5.79 ± 0.12 ^b^	7.69 ± 0.27 ^a^	4.98 ± 0.13 ^b^	0.001
Creatinine (μmol L^−1^)	97.20 ± 4.4 ^a^	97.2 ± 1.80 ^a^	97.2 ± 0.90 ^a^	0.30
Total bilirubin (μmol L^−1^)	4.28 ± 1.37 ^a^	4.45 ± 0.17 ^a^	4.28 ± 0.17 ^a^	0.90
ALP (U L^−1^)	198 ± 5.70 ^a^	99.1 ± 4.01 ^b^	99.8 ± 3.10 ^b^	0.001
NEFA (mmol L^−1^)	0.30 ± 0.005 ^b^	0.30 ± 0.01 ^b^	1.80 ± 0.05 ^a^	0.001
BHBA (mmol L^−1^)	0.30 ± 0.02 ^b^	0.99 ± 0.03 ^a^	0.96 ± 0.03 ^a^	0.001
Insulin (μIU mL^−1^)	5.10 ± 0.05 ^a^	5.01 ± 0.08 ^a^	4.9 ± 0.20 ^a^	0.70
T3 (pmol L^−1^)	152.20 ± 3.41 ^b^	155.3 ± 4.61 ^b^	272.8 ± 3.2 ^a^	0.001
T4 (pmol L^−1^)	7.08 ± 0.09 ^b^	7.21 ± 0.13 ^b^	9.14 ± 0.13 ^a^	0.001
IGF-1 (μg L^−1^)	3.20 ± 0.10 ^b^	7.1 ± 0.05 ^a^	3.3 ± 0.11 ^b^	0.001

Data are presented as mean ± SE. Means within the same row bearing different superscript letters (a–b) differ significantly (*p* < 0.05), with superscript “a” indicating the highest mean value among the compared groups. ALP: Alkaline phosphatase; NEFA: Non-Esterified Fatty Acids; BHBA: Beta-hydroxy-butyric acid; T3: Triiodothyronine; T4: Thyroxine; IGF-1: Insulin-like Growth Factor 1.

**Table 3:** Antioxidants and acute phase protein parameters in Shami goats during different physiological states (mean ± SE).

Parameters	Non Pregnant	Late Pregnant	Early Lactating	*p*-Value
GSH (mg dL^−1^)	40.90 ± 0.61 ^a^	20.7 ± 0.72 ^b^	20.5 ± 0.41 ^b^	0.001
GPx (U g Hb^−1^)	62.41 ± 1.40 ^a^	30.5 ± 0.51 ^b^	27.7 ± 0.70 ^b^	0.001
SOD (U mL^−1^)	66.40 ± 0.31 ^a^	32.8 ± 0.80 ^b^	32.7 ± 0.61 ^b^	0.001
Catalase (U L^−1^)	57.71 ± 1.0 ^a^	30.3 ± 0.31 ^b^	28.8 ± 0.32 ^b^	0.001
MDA (µmol L^−1^)	6.70 ± 0.08 ^b^	14.58 ± 0.20 ^a^	6.70 ± 0.10 ^b^	0.001
Hp (ng mL^−1^)	40.61 ± 0.10 ^c^	53.4 ± 0.41 ^b^	66.9 ± 0.08 ^a^	0.001
SAA (mg L^−1^)	4.40 ± 0.08 ^a^	4.5 ± 0.10 ^a^	4.5 ± 0.20 ^a^	0.90
Fb (g L^−1^)	4.40 ± 0.05 ^a^	4.5 ± 0.08 ^a^	4.7 ± 0.20 ^a^	0.70

Data are presented as mean ± SE. Means within the same row bearing different superscript letters (a–c) differ significantly (*p* < 0.05), with superscript “a” indicating the highest mean value among the compared groups. GSH: Glutathione reduced; GPx: Glutathione peroxidase; SOD: Superoxide dismutase; MDA: Malondialdehyde; Hp: Haptoglobin; SAA: Serum amyloid A; Fb: Fibrinogen.

## Data Availability

All original data presented in this paper are included in this paper. Any further questions can be sent to the corresponding authors.

## Abbreviations

APPs	Acute-phase proteins
RBC	Red blood cells
Hb	Hemoglobin
PCV	Packed cell volume
WBC	White blood cells
ALP	Alkaline phosphatase
NEFA	Non-Esterified Fatty Acids
BHBA	Beta-hydroxy-butyric acid
T3	Triiodothyronine
T4	Thyroxine
IGF1	Insulin-like Growth Factor 1
GSH	Glutathione reduced
GPx	Glutathione peroxidase
SOD	Superoxide dismutase
MDA	Malondialdhyde
Hp	Haptoglobin
SAA	Serum amyloid A
Fb	Fibrinogen
*SIRT1*	Sirtuin 1
*HMOX1*	Heme oxygenase 1
*SOD2*	Superoxide dismutase 2 (mitochondrial)
*PPARA*	Peroxisome proliferator-activated receptor alpha
*CPT1A*	Carnitine palmitoyltransferase 1A
*HMGCS2*	3-Hydroxy-3-methylglutaryl-CoA synthase 2
*CD36*	Cluster of differentiation 36
*LIPE*	Lipase E (hormone-sensitive lipase)
*PRKAA1*	Protein kinase AMP-activated catalytic subunit alpha 1
*SLC2A1*	Solute carrier family 2 member 1
*SLC2A4*	Solute carrier family 2 member 4
*NRF2*	Nuclear factor erythroid 2-related factor 2
*GPX1*	Glutathione peroxidase 1
*CAT*	Catalase
*TXN*	Thioredoxin
*HSP70*	Heat shock protein 70
*IL6*	Interleukin 6
*BECN1*	Beclin 1
*ATG5*	Autophagy-related 5
*GAPDH*	Glyceraldehyde-3-phosphate dehydrogenase
